# The Importance of the Position of Medical Examiner for Life Insurance1Read at the thirty-third annual meeting of the Medical Association of Central New York, held at Rochester, N. Y., October 16, 1900.

**Published:** 1901-01

**Authors:** Frank W. Maloney

**Affiliations:** Rochester, N. Y.


### THE IMPORTANCE OF THE POSITION OF MEDICAL EXAMINER FOR LIFE INSURANCE.[1]

By FRANK W. MALONEY, M. D., Rochester, N. Y.

IN BRINGING the subject of medico-insurance before you today, I do so with the idea that I might possibly set forth a few thoughts for the benefit of those of you who are interested as examiners for insurance companies, or those of you who are seeking an opportunity to act in that capacity.

The business of life insurance has developed so rapidly in this the closing of the century, and representing the aggregate number of risks in force at the present time in dollars, appalls the wildest flights

1. Read at the thirty-third annual meeting of the Medical Association of Central New York, held at Rochester, N. Y., October 16, 1900.

of the imagination. Billions is the word used now when expressing the vast interests of life insurance, over fifteen billion dollars of insurance being in force at the close of last year.

The medical examiner being a factor in this process, the question is, [1]Does the medical profession (or the class identified with insurance) fully appreciate the responsibility laid upon it by the business, or if appreciating it, does it measure up the requirements and fairly meet the obligation implied where such a vast sum of money is involved? In my opinion, I do not believe that the profession as a whole fully appreciates and considers the important position they occupy as the guardsmen of such vast financial interests. Why? Because they have not been taught the requirements of an examiner before they graduated. A large number of medical men when in practice a few years learn that the income and recognition derived from medico-insurance practice is very desirable, and here do they feel that they have the necessary qualifications to carry out and perform this kind of work.

What does an insurance company desire in its medical representative? They say, he must be a full-fledged physician. It is not enough that he be a graduate of a reputable medical college; he must have a fitness for the position. Companies ask a candidate to fill out a blank carefully devised to bring out the needed information as to his educational preparation. His name, age, date of graduation, and name of college, hospital experience, membership in societies, and has he had instruction in physical diagnosis, microscopy, bacteriology, urinary analysis, hygiene, and how long has he practised, and references from reputable physicians and business men in his community. Companies desire intellectual work and not mere routine duty. Their business is of vast interest and it will never be less than it is today, and therefore physicians trained in the allied sciences are needed. As insurance takes in everything, "it is a science of general averages, and probabilities enter largely into the business." A medical examiner should possess the diagnostic skill of a successful practitioner, combined with tact and judgment, as occasions will arise when such attainments will be very useful. If he conducts his practice in a careless manner, he is sure to do likewise in the examination of the applicant. An examiner must be a man of integrity; his reputation is at stake each time he makes out a medical report, as insurance is a legal business and governed by the laws of the state, which renders him liable to be questioned concerning the

---

1. The "Medical Examiner," June, 1897.

statements he makes over his own signature. This phase is often overlooked in examinations.

The examiner performs his work for pay and it is paid for; therefore, it is a commercial transaction and false pretenses have no more place here than anywhere else. Physicians are bound to perform their duties here as in any other branch of their work, that is, to make examinations according to the principles and practices which prevail among medical men generally. That is the rule which the law holds them to in general practice, and that is the rule which the court would lay down if such questions were brought before it.

How few practitioners are competent to make a thorough examination of an applicant. A writer in the *University Medical Magazine*, of Philadelphia, says that only 20 per cent. of physicians in private practice make urinary examinations or know how to make them. This fact, if true, is deplorable, and in the present day of exact sciences it demonstrates slip-shod methods on the part of the faculties of colleges.

It should be the aim of practising physicians to fit themselves for examiners for life insurance companies, as it will be the means of getting better known and meeting individuals with whom they would otherwise never have come in contact, and this acquaintance under auspices which naturally makes an impression upon those examined.

We come now to the examination of an applicant. The first impression one gets of an individual is certainly a very valuable one, and should not fail to be noted on the examination paper. Does he appear to be a man who would indicate resistance to fatigue; does he look strong and healthy? We know that there are some individuals who, upon most careful examination, show nothing whatever the matter with them, but yet there is something about them that impresses one that they will not be long-lived. Is this the case with the applicant? Does he look as if he was worrying about business cares? Does he look as if he took care of himself? Does he eat his meals quietly and give the food time to digest? It is this very impression in one accustomed to making a number of examinations that is as important as his power to detect disease, and frequently upon the closest examination the examiner will not be able to detect disease in an individual, yet he is impressed with the fact that he has not a healthy appearance.

It is the detection of this want of power of resistance to disease on the part of the applicant that constitutes the best class of examiners. We know that the great majority of men of the present day, who in the mad rush for wealth and existence cannot long stand the strain and tear upon the system to which they submit themselves. There are some men who can stand it because they have a strong and healthy constitution, but, by far, the majority of cases succumb to disease before the expectation of life.

The examination should begin by a study of the applicant as a whole, by noting the color of his eyes, his complexion. Does his skin show a healthy hue? are his eyes bright? does his face exhibit the appearances of health, not drawn by disease or lined with wrinkles, which denotes premature ageing? Note the color of the hair and examine the tongue, and see whether there is any evidence by it of disease or disorder of the digestive tract, and include in this the throat for ulcers, patches, chronic pharyngitis, enlargement of the tonsils, or any indications which would point to cancer or syphilis. Is the eye bright? are the pupils equal? have they the appearance of sleepiness or drowsiness, to denote the use of narcotics? Is the tongue thickly coated or eyes bloodshot? the odor of the breath like the peculiar odor of drinkers. Then the appearance of the skin: Is there eruption or the cachexiæ of the various diseases? yellowness or anemic condition? puffiness about the eyes? pallor, blueness of lips, prominent cheek-bones with the hectic of phthisis? and during the conversation the applicant's voice and the rapidity of the breath should be noted. The habits of the applicant have an important bearing on life insurance. Intemperance in the use of alcoholic drinks is a fruitful source of disease: we see this in the condition of the nervous system, the eye, the tongue, and also with excessive beer drinking. Tobacco in excess produces a disturbance in the nervous system, causing rapid pulse, palpitation of the heart, loss of appetite and emaciation.

The pulse, as a rule, is indicative of the heart, but at times, owing to the atheromatous condition of the vessels, the pulse wave may distinctly differ from the cardiac impulse. A slight intermittency may be noted in the pulse which would escape one's attention upon examination of the heart. The sphymograph is used to a decided advantage.

The heart should be examined before the chest in general, because the circulation is excited by the increased respiration during the examination of the lungs. Palpitation, percussion and auscultation will give the desired knowledge of the cardiac sounds, showing the different impulses, irregularity of heart's action.

The applicant should be divested of his coat and vest during the physical examination. In measuring the chest, the tape should be placed at about the third costo-sternal junction, so as to avoid the mammary glands, and the waist measure taken on a line with the umbilicus. By lifting up the shirt and underwear, on inspection an idea is obtained of the vital functions of the heart and lungs, the apex beat is noted, the shape of the lungs and deformities, if any, the rate of respiration, the degree of inspiration and expiration.

Next, by percussion we note the various sounds in all parts of the chest, and the examiner knowing the normal, those of disease he would readily distinguish. By auscultation, either with the ear or stethoscope, the various conditions of the respiratory sounds are heard.

The man who knows he is not up to par feels the necessity of insurance, but the phthisical man imagines himself improving in health until the time of his death. He will conceal the fact that he has had hemorrhages, morning cough, emaciation and shortness of breath. Here the thermometer, special attention to the apices, the family history, and in suspected cases examination of the sputum for tubercle bacilli.

In the examination of the abdomen the examiner should make use of inspection, palpation, percussion and auscultation, as with the lungs and chest. The liver should be examined while standing for evidence of tenderness, or bulging and enlargement or atrophy. The spleen in like manner. The bowels should be palpated, also the bladder, genito-urinary organs and rectum,—the rectum for hemorrhages, fistula and cancer. In the examination of the urine (every medical examiner should be fu ly posted as to the testing of the urine), the first important test is for albumin, and second that for sugar, because evidences of Bright's disease and diabetes can be detected years in advance of the disease. Here the microscope will demonstrate the finer tests, such as casts, urea, pus and oxylate of lime. Finally the nervous system. Here slowness of speech sentences broken off or uncertain, efficiency in memory, agitated lips, tremulous tongue, double sight and step defective. Here also evidences of locomotor ataxia must be looked for, neuralgias, headaches, vertigo, syphilis and nervous prostration.

With the examination of women, special attention should be given to the breast and the abdominal organs, the menstrual changes and child-bearing.

And now, after the applicant has signed the report, the examiner's best judgment, his honesty and his integrity is brought to meet the summary. Is the applicant a first-class risk? Is he a good risk, or only a fair risk? Here the golden rule must be remembered.

Personal friendship, fraternal ties, professional relations or sympathetic interests, are not to be considered if the examiner is an honest man. His rating should be according to the dictates of his conscience, and when he allows an applicant to pass as a first-class risk when he knows the applicant is not one, that examiner underestimates the importance of his work, his private practice will run accordingly vascillating.

There is a great deal to be said on this very important subject, but the limits of a paper of this kind cannot encompass it all. I would, therefore, in conclusion earnestly ask the members here present who are associated with the colleges of Buffalo and Syracuse, to use their best endeavors with the faculties that at least two lectures be given to the graduating class on the subject of examining for life insurance companies, and a thorough course in physical diagnosis. We know that owing to the importance of this kind of work life insurance companies demand that their examiners be in practice not less than three years, and some of the companies make five years the limit. It is a sad commentary on the ability and honesty of our profession when one company divides its insurance into two classes, as an experiment, keeping and noting the record of those examined and those unexamined, to find after a certain number of years that the mortality of those unexamined was less than that of the examined. This blot on the profession can only be erased by the colleges teaching their graduates the importance of this kind of work. A thorough medical examiner will make a successful practitioner.

332 West Avenue.

